# Homicide Rates Among Persons Aged 10–24 Years — United States, 1981–2010

**Published:** 2013-07-12

**Authors:** Corinne David-Ferdon, Linda L. Dahlberg, Scott R. Kegler

**Affiliations:** Div of Violence Prevention; Div of Analysis, Research, and Practice Integration, National Center for Injury Prevention and Control, CDC

Homicide disproportionately affects persons aged 10–24 years in the United States and consistently ranks in the top three leading causes of death in this age group, resulting in approximately 4,800 deaths and an estimated $9 billion in lost productivity and medical costs in 2010 (1). To investigate trends in homicide among persons aged 10–24 years for the period 1981–2010, CDC analyzed National Vital Statistics System data on deaths caused by homicide of persons in this age group and examined trends by sex, age, race/ethnicity, and mechanism of injury. This report describes the results of that analysis, which indicated that homicide rates varied substantially during the study period, with a sharp rise from 1985 to 1993 followed by a decline that has slowed since 1999. During the period 2000–2010, rates declined for all groups, although the decline was significantly slower for males compared with females and for blacks compared with Hispanics and persons of other racial/ethnic groups. By mechanism of injury, the decline for firearm homicides from 2000 to 2010 was significantly slower than for nonfirearm homicides. The homicide rate among persons aged 10–24 years in 2010 was 7.5 per 100,000, the lowest in the 30-year study period. Primary prevention strategies remain critical, particularly among groups at increased risk for homicide.

National homicide counts and population estimates for U.S. residents were obtained from the National Vital Statistics System using CDC’s Web-based Injury Statistics Query and Reporting System (WISQARS) for persons aged 10–24 years for the period 1981–2010 (1,2). Data were stratified by year, sex, 5-year age group (i.e., 10–14, 15–19, and 20–24 years), and mechanism of injury (i.e., firearm or nonfirearm). Homicide counts and population estimates were further stratified by race/ethnicity for 1990–2010 (i.e., non-Hispanic white, non-Hispanic black, non-Hispanic other, and Hispanic).* Annual homicide rates (per 100,000 population) were determined overall and for the indicated strata. The most recent period (2000–2010) is of particular interest because it best reflects the populations currently at highest risk for whom the continued implementation of prevention strategies remains crucial. Trends for this later period were analyzed using a negative binomial rate regression modeling approach, allowing formal statistical evaluation of trends and comparisons across strata.

The overall homicide rate among persons aged 10–24 years varied substantially during the 30-year study period (Figure 1). Rates rose sharply from 1985 to 1993, increasing 83%, from 8.7 per 100,000 in 1985 to 15.9 in 1993. From 1994 to 1999, the overall rate declined 41%, from 15.2 per 100,000 in 1994 to 8.9 in 1999. Modeled rates indicate a slow but statistically significant downward trend in homicide in this age group for the period 2000–2010 (p=0.04), with a model-estimated decline of approximately 1% per year. The overall homicide rate in 2010 (7.5 per 100,000) was the lowest rate during the 30-year period. Nearly 80% of all homicides during the 30-year study period were firearm homicides (79% overall; range of annual percentages: 64%–85%). The annual rate of firearm homicide was on average 3.7 times the annual rate of nonfirearm homicide during this period. Among persons aged 10–24 years, males, those aged 20–24 years, and blacks had the highest rates of homicide over the 30 years examined (Figures 2 and 3). In 2010, the homicide rates for these groups were 12.7 per 100,000 for males, 13.2 for persons aged 20–24 years, and 28.8 for blacks.

Patterns in homicide rates among persons aged 10–24 years for the period 2000–2010 were further examined by sex, age group, race/ethnicity, and mechanism of injury. Homicide rates for males remained substantially higher than rates for females (Figure 2). Although model-estimated rates for males and females indicate declines, in relative terms, the decline for males was significantly slower than the decline for females (p=0.03). When homicide rates were examined by age group, rates for persons aged 20–24 years remained highest, and rates for persons aged 10–14 years remained lowest (Figure 2). Model-estimated rates indicate declines for all three age groups. Age-specific declines in homicide rates were not found to be significantly different.

The examination of homicide rates by race/ethnicity for the period 2000–2010 shows that rates for blacks aged 10–24 years remained the highest and rates for whites in this age group remained the lowest (Figure 3). Model-estimated rates indicate a decline for all four racial/ethnic groups. The decline in homicide rates for blacks was significantly slower than the declines for Hispanics and persons of other racial/ethnic groups (p<0.01). The decline for blacks also was slower than the decline for whites, but the difference was not significant. Model-estimated rates indicate a decline during 2000–2010 for both firearm and nonfirearm homicides, with the decline for the firearm homicide rate significantly slower than the decline for the nonfirearm homicide rate (p<0.01) (Figure 1).

## Editorial Note

For the past three decades, homicide has been a leading cause of death among adolescents and young adults in the United States. The findings in this report demonstrate that homicide rates among persons aged 10–24 years varied substantially over time but showed a decline from 1994 through 2010. Changes in the overall homicide rate for this age group during the 30-year study period primarily reflect variations in homicide rates for the groups at highest risk (i.e., males, persons aged 20–24, and blacks). These findings highlight the fact that despite an overall decline in homicide to a 30-year low in 2010, some adolescents and young adults remain disproportionately affected, and more recent declines in rates have been slower for those at increased risk for homicide. Overall, the findings of this report demonstrate that progress has been made in reducing homicide in these populations, but progress is slowing, and primary prevention of violence in these populations needs continued emphasis.

The variability of homicide rates among persons aged 10–24 years over time is similar to trends for other violent crime rates (3). Previous research has linked the rise and subsequent decline in homicide and violent crime in this population to changes in drug use and drug-related crime, shifting community demographics, community-based and problem-oriented policing (i.e., identification and analysis of a specific type of crime to develop customized, coordinated, and improved community response strategies), and varying economic conditions (4). Focused deterrence strategies specifically address serious violence and crime, and when implemented well, these strategies show promise in reducing crime though more rigorous evaluations are needed (5). Focused deterrence approaches vary in design and generally include an interagency coalition (e.g., law enforcement and social service providers), identification of crime perpetrator groups (e.g., gang members), communication of incentives (e.g., avoidance of incarceration and availability of education and employment services) to these groups to stop them from continuing to engage in violence, and law enforcement and social service organizations implementing activities (e.g., vocational training, mentoring, housing assistance, and substance use treatment) directed toward these groups.

Although law enforcement responses to violence and focused attention on high crime areas and perpetrators help to reduce the continuation of violence, they do not stop violence from happening in the first place. Research on youth violence demonstrates the importance of implementing primary prevention approaches that begin in childhood to disrupt the developmental pathways to serious violence in adolescence and adulthood and can be diffused across large populations (6,7). A number of primary prevention strategies are scientifically proven to reduce the risk for and occurrence of youth violence and provide critical complements to law enforcement approaches (6,7). Examples of primary prevention strategies include 1) school-based programs that build the communication skills of youths to nonviolently solve problems; 2) family approaches that help caregivers set age-appropriate rules and effectively monitor children’s activities and relationships; and 3) policy, environmental, and structural approaches that enhance safety and increase opportunities for positive social interaction. For example, innovative community-level strategies, such as business improvement districts, address socioeconomic and other factors that influence rates of violence, and initial results show that these approaches contribute to significant reductions in rates of crime and violence and cost savings attributed to such reductions, fewer arrests, and lower prosecution-related expenditures (8). Many other prevention strategies have been shown to reduce the risk for youth violence and result in a significant return on investment (7).

The findings of this report are subject to at least two limitations. First, race and ethnicity were not coded separately until 1990, restricting examination of racial/ethnic group statistics and differences to the period 1990–2010. Second, comparisons of census self-report and death certificate reports of race and ethnicity show misclassification for Hispanics, Asian/Pacific Islanders, and American Indian/Alaska Natives, which might result in underestimation of rates for these groups (9).

Community-wide and long-lasting reductions in youth violence come from comprehensive approaches that include multiple evidence-based strategies and collaboration of diverse groups, such as public health, justice, education, businesses, and community groups (7). The public health sector brings to this collaboration a science-driven approach that focuses on primary prevention and promotion of population-wide health and safety. CDC’s Academic Centers of Excellence in Youth Violence Prevention and the Striving To Prevent Youth Violence Everywhere national initiative are examples of collaborative approaches to strategically plan and implement comprehensive, evidence-based strategies that include the public health sector (10).

What is already known on this topic?

Homicide consistently ranks in the top three leading causes of death among persons aged 10–24 years in the United States.

What is added by this report?

Youth homicide rates during 1981–2010 fluctuated widely over time but had a downward trend beginning in 1994. The 2010 youth homicide rate of 7.5 per 100,000 is the lowest rate in the 30 years examined. However, the decline in overall youth homicide rates has slowed in the last decade. Declines have been slower for the highest-risk groups (e.g., males and non-Hispanic blacks) and for firearm homicide.

What are the implications for public health practice?

The continued use of evidence-based, primary prevention strategies to stop youth violence is needed. The public health sector reaching the highest-risk youths with effective prevention strategies is particularly critical.

## Figures and Tables

**FIGURE 1:**
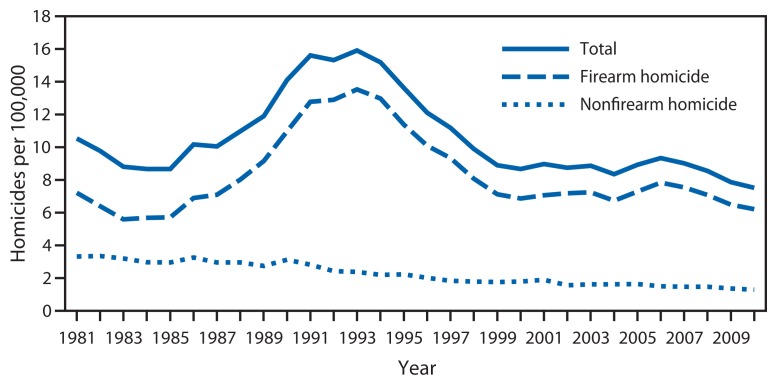
Firearm and nonfirearm homicide rates among persons aged 10–24 years — United States, 1981–2010

**FIGURE 2:**
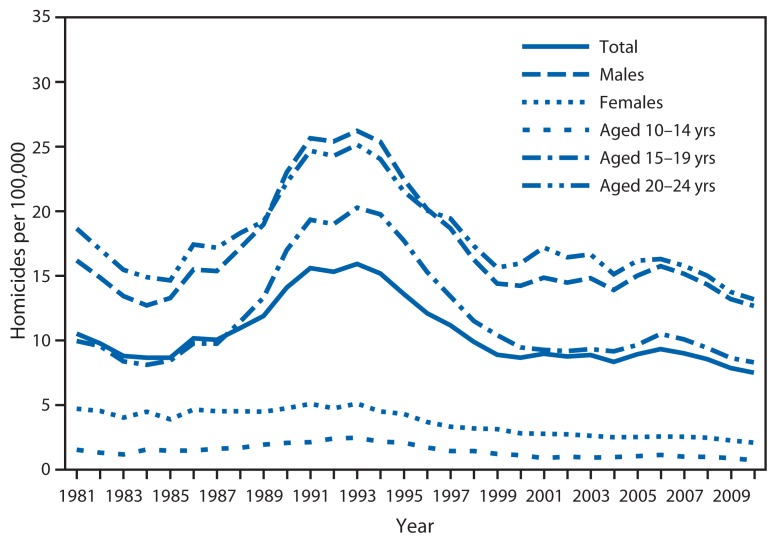
Homicide rates among persons aged 10–24 years, by sex and age group — United States, 1981–2010

**FIGURE 3:**
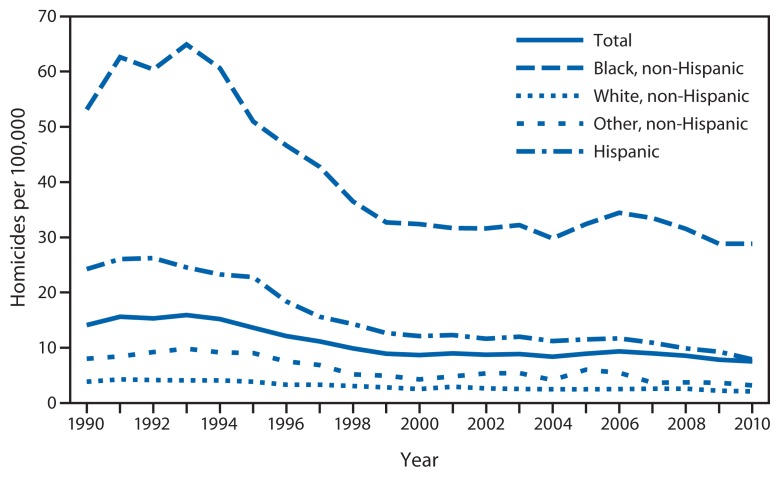
Homicide rates among persons aged 10–24 years, by race/ethnicity — United States, 1990–2010

## Footnotes

* Unless indicated otherwise, all racial/ethnic groups are non-Hispanic; Hispanics can be of any race. For this report, other races include Asian/Pacific Islander and American Indian/Alaska Native.
